# Reduced Racial Disparity as a Result of Survival Improvement in Prostate Cancer

**DOI:** 10.3390/cancers15153977

**Published:** 2023-08-04

**Authors:** Baoyi Zhang, Jianrong Li, Mabel Tang, Chao Cheng

**Affiliations:** 1 Department of Chemical and Biomolecular Engineering, Rice University, Houston, TX 77030, USA; bz26@rice.edu; 2 Department of Medicine, Baylor College of Medicine, Houston, TX 77030, USA; jalen.li@bcm.edu; 3 Department of Biosciences, Rice University, Houston, TX 77030, USA; mst6@rice.edu; 4 Dan L Duncan Comprehensive Cancer Center, Baylor College of Medicine, Houston, TX 77030, USA; 5 The Institute for Clinical and Translational Research, Baylor College of Medicine, Houston, TX 77030, USA

**Keywords:** prostate cancer, racial disparity, socioeconomic disparity, prognosis

## Abstract

**Simple Summary:**

The survival rates of prostate cancer substantially vary by race and socioeconomic status. Compared with non-Hispanic white men, African American men tend to have a two-fold increased risk of death. Similarly, inferior survival outcomes are found in low-income patients compared to high-income patients. Although such disparities have been extensively investigated in past years, there has still been a lack of studies investigating the trends in these disparities in recent decades. Hence, we analyzed the Surveillance Epidemiology End Results (SEER) data containing 534,076 prostate cancer patients diagnosed from 2004 to 2018. Our study revealed that the racial disparity fluctuated during 2004–2011, following which it substantially reduced. In contrast, the socioeconomic disparity witnessed a consistent increase during 2004–2018. Our findings suggested more efforts were needed to improve the treatment plans for patients with limited financial resources, which may also aid in updating the existing treatment guidelines for prostate cancer.

**Abstract:**

Prostate cancer is a cancer type associated with a high level of racial and socioeconomic disparities as reported by many previous studies. However, the changes in these disparities in the past two decades have not been systematically studied. In this study, we investigated the Surveillance Epidemiology End Results (SEER) data for prostate cancer patients diagnosed during 2004–2018. African Americans and Asians showed significantly better and worse cancer-specific survival (CSS), respectively, compared to non-Hispanic white individuals after adjusting for confounding factors such as age and cancer stage. Importantly, the data indicated that racial disparities fluctuated and reached the highest level during 2009–2013, and thereafter, it showed a substantial improvement. Such a change cannot be explained by the improvement in early diagnosis but is mainly driven by the differential improvement in CSS between races. Compared with Asians and non-Hispanic whites, African American patients achieved a more significant survival improvement during 2014–2018, while no significant improvement was observed for Hispanics. In addition, the SEER data showed that high-income patients had significantly longer CSS than low-income patients. Such a socioeconomic disparity was continuously increasing during 2004–2018, which was caused by the increased survival benefits of the high-income patients with respect to the low-income patients. Our study suggests that more efforts and resources should be allocated to improve the treatment of patients with low socioeconomic status.

## 1. Introduction

Prostate cancer is the most common male cancer and the second-leading cause of male cancer deaths in the USA, accounting for 27% of all male cancers and 11% of cancer deaths in men in 2022 [1]. While the overall incidence remains stable, the proportion of prostate cancer diagnosed at an advanced stage has continued to increase by 4–6% per year in the past decade [1].

Racial disparities in various cancer types have been extensively investigated in many previous studies [2,3,4,5,6,7,8,9,10,11]. Compared with non-Hispanic white men, African American males are associated with 1.6-fold-increased age-adjusted incidence and 2.1-fold-increased mortality in prostate cancer [2,12,13]. In contrast, Asian males are associated with lower prostate cancer incidence and decreased mortality from the disease [14,15,16]. Such disparities among different races might be explained by many factors, including genetic variations, environmental exposures, socioeconomic status, access to and quality of healthcare, etc. [17,18,19,20]. For example, it has been reported that African American males are less likely to access prostate cancer screening and high-quality care, and as a consequence, are more likely to be diagnosed at an advanced stage of prostate cancer and receive inadequate treatment regimens [2].

In addition to racial disparities, socioeconomic status has also contributed to the incidence of prostate cancer and the mortality of patients with prostate cancer [21,22]. In general, males with higher socioeconomic status are more likely to be diagnosed with prostate cancer [23,24], but at nonadvanced stages and low grades [25,26]. Moreover, prostate cancer patients with favorable socioeconomic status tend to have lower mortality [25,27]. Such socioeconomic disparities can be explained by unequal access to health and screening services, as well as other factors influenced by socioeconomic status [22]. Annual income is one of the most impactful socioeconomic factors. In general, patients with a lower income are less likely to have higher education and more likely to be unemployed and uninsured/underinsured. It has been reported that low-income prostate cancer patients tend to be diagnosed at an advanced stage and are associated with poor survival [21,28].

Although the racial and socioeconomic disparities in prostate cancer survival have been well studied, there has been a lack of investigations into the trends in these disparities in the past decades. In this study, we sought to quantify the changes in racial and socioeconomic disparity in prostate cancer over time. We analyzed the Surveillance Epidemiology End Results (SEER) prostate cancer data [29] from 2004 to 2018 to investigate the two types of disparities in different time periods while adjusting for established prognostic factors. In addition, to understand the underlying reasons behind such changes, we explored potential contributing factors such as alterations in age and stage at diagnosis.

## 2. Materials and Methods

In this study, we analyzed the SEER data [29] (accessed on 6 March 2022) for prostate cancer patients diagnosed between 2004 and 2018. The major patient clinical information from SEER includes (1) the year of diagnosis, (2) the age at diagnosis, (3) the prostate cancer stage (I, II, III, and IV), (4) the race and origin recode (NHW, NHB, NHAIAN, NHAPI, and Hispanic), and (5) median household income inflation adj to 2019. The prostate cancer stage of patients was defined based on the “Derived AJCC Stage Group, 6th ed (2004–2015)” [30], “Derived SEER Cmb Stg Grp (2016–2017)” [31], and “Derived EOD 2018 Stage Group (2018+)” [32] for patients diagnosed in 2004–2015, 2016–2017, and 2018, respectively. The overall and prostate-cancer-specific survival information was determined based on “Vital status recode (study cutoff used)”, “SEER cause-specific death classification”, and “Dead (attributable to this cancer dx)” provided by the SEER data.

To ensure high data quality and robustness in the analysis, we selected prostate cancer patients using the following criteria: (1) the “Type of Reporting Source” is from “Hospital inpatient/outpatient or clinic”, (2) the patient is only diagnosed to have prostate cancer (i.e., the variable “Sequence number” is labeled as “One primary only”), (3) less than 10% of the clinical variables have missing values, and (4) the age of diagnosis is between 40 and 85. The race of patients was determined based on the variable “Race and origin recode (NHW, NHB, NHAIAN, NHAPI, Hispanic)” provided by SEER. According to this variable, “Non-Hispanic White”, “Non-Hispanic Black”, “Non-Hispanic Asian or Pacific Islander”, and “Hispanic (All Races)” were categorized as non-Hispanic white, Black, Asian, and “Hispanic”, respectively. The final data contained a total of 534,076 unique patients (Figure S1, Table S1).

### Survival Analysis

Survival analyses were performed by using the R package “survival”. Multivariate Cox proportional hazards regression was performed to calculate the hazard ratios (HRs) and 95% confidence intervals (CIs) of the interested variables’ association with prostate-cancer-specific or overall survival. Known prognostic factors such as age, race, tumor stage, lymph node involvement, distal metastasis, PSA levels, Gleason scores, and income were selected as confounding variables. Of note, PSA levels and Gleason scores were only available for patients diagnosed after 2009. For each multivariable analysis, we excluded variables with missing values. To estimate the overall racial and socioeconomic disparities, “Age at diagnosis” and “Median household income” were converted into categorical variables in the following ways. The “Age at diagnosis” was stratified into 40–55 (Younger), 56–70 (Middle), and 71–85 (Older) groups; the “Median household income inflation adj to 2019” was regrouped into “<USD60,000” (Low), “USD60,000–USD74,999” (Intermediate), and “>USD75,000” (High), with comparable patient numbers. To determine the racial disparities, non-Hispanic white patients were used as the baseline. To determine the socioeconomic disparities, the low-income patients (adjusted income < USD 60,000) were selected as the baseline. To determine the improvement in patient survival, the patients diagnosed between 2004 and 2008 were used as the baseline. To determine the risk between different cancer stages, stage I was selected as the baseline.

We selected age, stage at diagnosis, race, and median household income for stratification analyses. These variables were selected based on (1) known prognostic values, (2) less than 10% missing values, and (3) <95% of the most frequent values for categorical variables. In the stratified analyses, the original variable types and values were used in the Cox regression models to ensure the best statistical power. Namely, “Age at diagnosis” was included as a continuous variable, while “Median household income” was included as a variable with 10 categories (originally defined by SEER).

## 3. Results

### 3.1. Overall Racial and Socioeconomic Disparities

**Figure 1:**
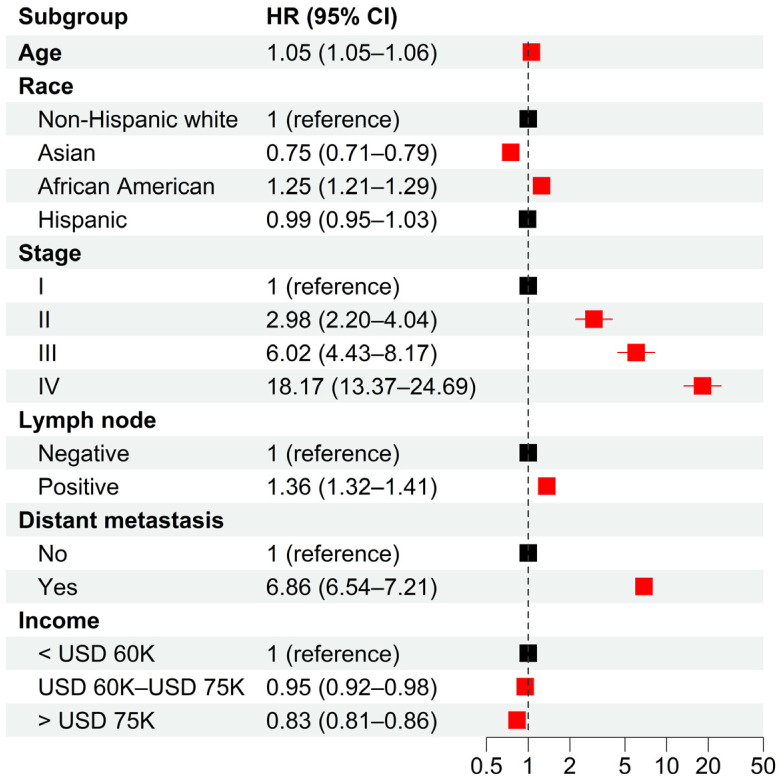
**Hazard ratios of clinical characteristics for cancer-specific death in 2004–2018 from multivariable Cox models.** Multivariable Cox models were adjusted for age, race, tumor stage, lymph mode involvement, distant metastasis, and income. In paratheses, 95% CIs are presented. Red color indicates a significance level of *p* < 0.05.

From the SEER database, we identified a total of 534,076 prostate cancer patients diagnosed between 2004 and 2018. In total, 67.5%, 5.0%, 16.2%, and 10.0% of the patients were non-Hispanic white, Asian, African American, and Hispanic, respectively. Multivariable analysis indicated significant racial disparities in cancer-specific survival (CSS) (Figure 1). Compared to non-Hispanic white patients, Asians had significantly longer CSS (adjusted HR = 0.75 with 95% CI = [0.71, 0.79]), while African Americans had significantly shorter CSS (adjusted HR = 1.25 with 95% CI = [1.21, 1.29]). There was no significant survival difference between Hispanic and non-Hispanic white patients. Furthermore, the analysis also indicated significant socioeconomic disparities, with intermediate- (adjusted HR = 0.95, 95% CI = [0.92, 0.98]) and high-income (adjusted HR = 0.83, 95% CI = [0.81, 0.86]) patients having significantly longer CSS than low-income patients. As expected, older age and later stage of diagnosis were associated with poor CSS. These results remained valid when PSA levels and Gleason scores were adjusted for patients diagnosed after 2009 (Figure S2A). Similar results were obtained when the overall survival (OS) of patients was investigated (Figure S2B,C).

### 3.2. Racial Disparities in Different Subgroups of Prostate Cancer Patients

**Figure 2:**
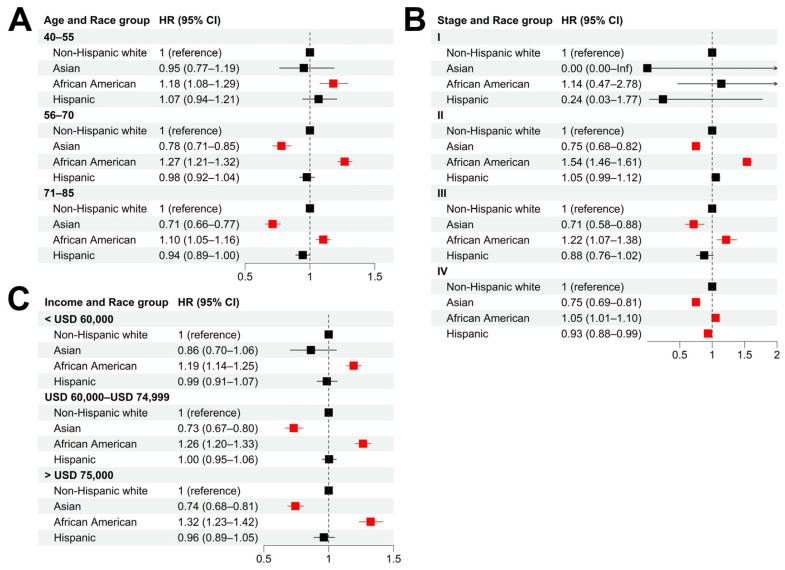
**Racial disparities in different patient subgroups.** (**A**–**C**): Hazard ratios of race for cancer-specific death in patient subgroups stratified by age (**A**), tumor stage (**B**), and income (**C**) using non-Hispanic white as baseline. Multivariable Cox models were adjusted for age, race, tumor stage, lymph mode involvement, distant metastasis, and income. In paratheses, 95% CIs are presented. Red color indicates a significance level of *p* < 0.05.

We performed stratified analyses to evaluate the influence of age (at the time of diagnosis), cancer stage, and income on the racial disparity of cancer survival. In all the age groups, African American patients showed significantly shorter CSS compared with non-Hispanic whites (Figure 2A and Figure S3A). The highest disparity was observed in the 56–70 age group (adjusted HR = 1.27, 95% CI = [1.21, 1.32]), compared with adjusted HR = 1.18 (95% CI = [1.08, 1.29]) for the 40–55 group and HR = 1.10 (95% CI = [1.05, 1.16]) for the 71–85 group. When stratified by cancer stage, African American patients showed significantly shorter CSS than non-Hispanic whites, with the most significant difference in CSS being observed in stage II patients (adjusted HR = 1.54, 95% CI = [1.46, 1.61]) (Figure 2B and Figure S3B). In all the income groups, African American patients had significantly shorter CSS than non-Hispanic whites (Figure 2C and Figure S3C).

Compared with non-Hispanic white patients, Asians were more likely to have longer CSS in different age groups (except for the 40–55 group) (Figure 2A and Figure S3A), stage groups at the time of diagnosis (except for stage I) (Figure 2B and Figure S3B), and income groups (except for the low-income group) (Figure 2C and Figure S3C). In most of the stratified groups, Hispanic patients did not have significant CSS differences from their non-Hispanic white counterparts, except in the stage IV group (adjusted HR = 0.93, 95% CI = [0.88, 0.99]).

### 3.3. Socioeconomic Disparities in Different Subgroups of Prostate Cancer Patients

**Figure 3:**
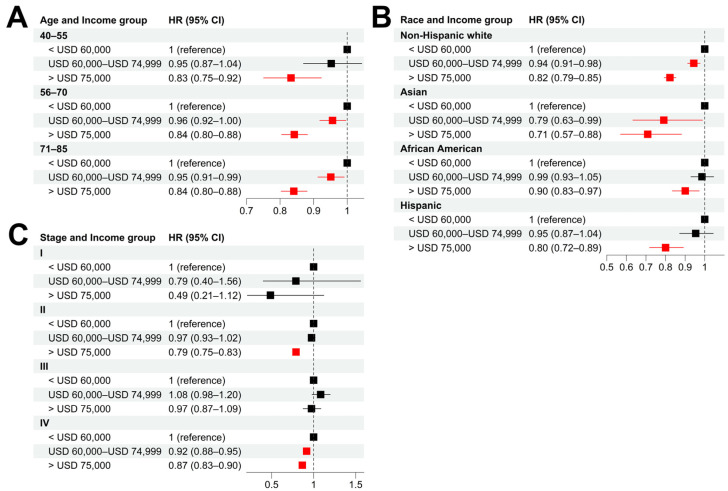
**Socioeconomic disparities in different patient subgroups.** (**A**–**C**): Hazard ratios of income for cancer-specific death in patient subgroups stratified by age (**A**), race (**B**), and tumor stage (**C**) using low-income (<USD 60,000) group as baseline. Multivariable Cox models were adjusted for age, race, tumor stage, lymph mode involvement, distant metastasis, and income. In paratheses, 95% CIs are presented. Red color indicates a significance level of *p* < 0.05.

Stratified analyses were also performed to evaluate the influence of age, cancer stage, and race on the socioeconomic disparity of cancer survival. As shown in Figure 3A,B and Figure S4A,B, patients with high income tended to have significantly longer CSS than those with low income in all the age and race groups. The same trend was observed in patients with stage II and IV prostate cancer at the time of diagnosis (Figure 3C and Figure S4C).

### 3.4. The Change in Racial and Socioeconomic Disparities from 2004 to 2018

**Figure 4:**
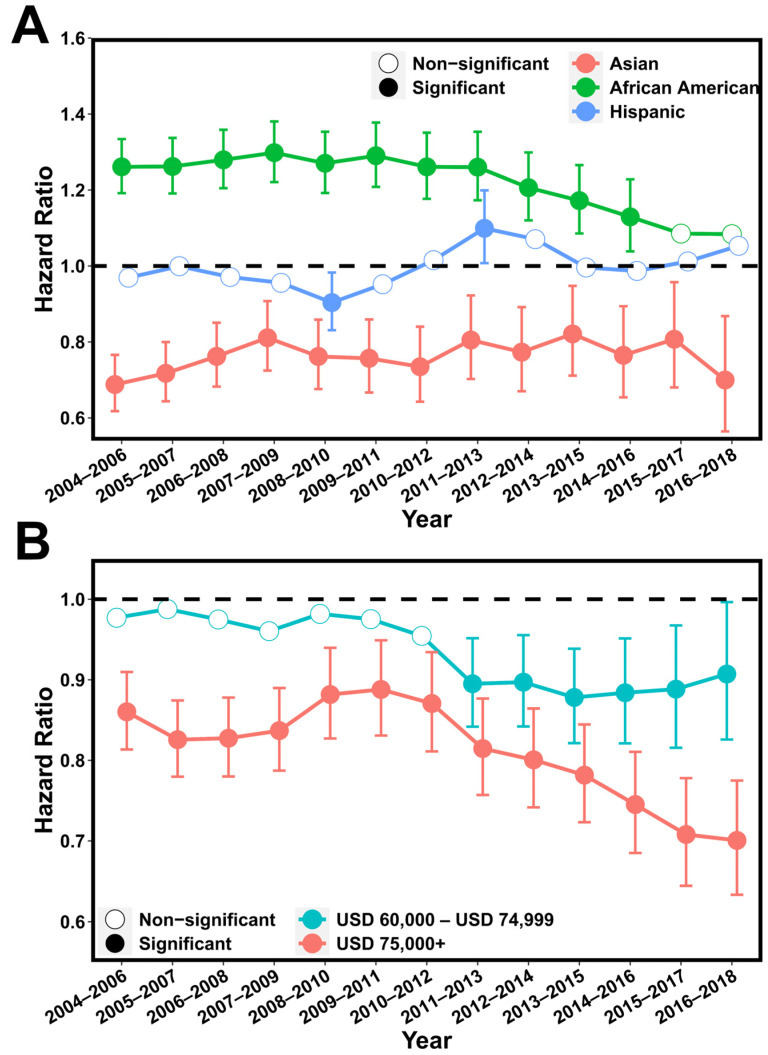
**The change in racial and socioeconomic disparity from 2004 to 2018.** (**A**): Hazard ratio of Asians, Blacks, and Hispanics for cancer-specific death using non-Hispanic whites as baseline. Multivariable Cox models were adjusted for age, tumor stage, lymph mode involvement, distant metastasis, and income. (**B**): Hazard ratio of intermediate-income (USD 60,000–USD 74,999) and high-income (>USD 75,000) groups for cancer-specific death using low-income (<USD 60,000) group as baseline. Multivariable Cox models were adjusted for age, race, and tumor stage. *p* values below 0.05 were considered significant.

Next, we investigated the changes in racial and economic disparity in each of the 3-year continuums from 2004 to 2018. Our results indicated that racial disparity fluctuated from 2004 to 2011 and became less serious after then (Figure 4A and Figure S5A). Specifically, African American patients had an adjusted HR = 1.30 (95% CI = [1.22, 1.38]) in the period of 2007–2009 that was reduced until the most recent period, 2016–2018, when compared with non-Hispanic whites. A similar trend was also observed for Asian patients. Hispanic patients showed significantly shorter CSS than non-Hispanic whites in the periods 2011–2013 and 2012–2014, but it was not significant in other periods. In contrast, the socioeconomic disparity has continuously increased over the past 15 years, with the high-income patients displaying significantly longer CSS compared with low-income patients in all 3-year periods (Figure 4B and Figure S5B). In the most recent period, 2016–2018, the highest disparity was observed, with an adjusted HR = 0.70 (95% CI = [0.63, 0.77]) for the high-income patients compared with the low-income patients.

### 3.5. The Change in Age and Cancer Stage at Diagnosis in Different Race and Income Groups

To understand the potential factors that explain racial and socioeconomic disparities in patient survival, we compared the average age and cancer stage at the time of diagnosis between different race groups and income groups. Interestingly, compared with non-Hispanic white patients, African Americans were more likely to be diagnosed at a younger age, while Asians were more likely to be diagnosed at an older age (Figure S6A). In addition, we observed no difference in the age at diagnosis among the three income groups (Figure S6B). Compared with non-Hispanic whites, the other races (African Americans, Asians, and Hispanics) were more likely to be diagnosed with metastatic prostate cancer (Stage IV), which is associated with a substantially worse prognosis than earlier stages (Stage I–III) (Figure S6C). When different income groups were compared, the intermediate-income group showed the highest rate of stage IV diagnosis, followed by the high-income group (Figure S6D).

### 3.6. The Survival of African American Patients Has Improved Rapidly in Recent Years

**Figure 5:**
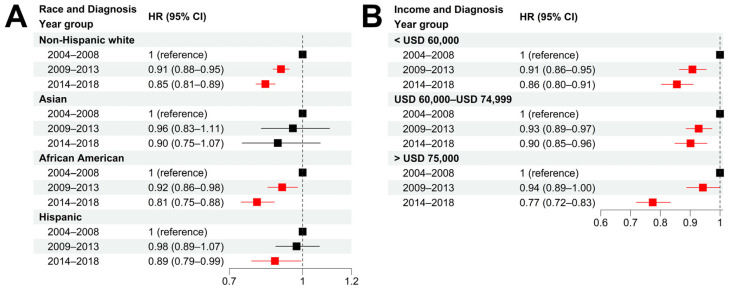
**Survival improvements in different race and socioeconomic groups.** (**A**,**B**): Hazard ratios of diagnosis year for cancer-specific death in patient subgroups stratified by race (**A**), and income (**B**) using non-Hispanic white and low-income (<USD 60,000) group as baseline, respectively. Multivariable Cox models were adjusted for age, race, tumor stage, lymph mode involvement, distant metastasis, and income. In paratheses, 95% CIs are presented. Red color indicates a significance level of *p* < 0.05.

We further examined the CSS improvement in different races and income groups in the past two 5-year periods (2009–2013 and 2014–2018) with respect to the period 2004–2008 (the year of diagnosis). Non-Hispanic white patients experienced a significant CSS improvement in both 5-year periods with an adjusted HR = 0.91 (95% CI = [0.88, 0.95]) for 2009–2013 and an adjusted HR = 0.85 (95% CI = [0.81, 0.89]) for 2014–2018, respectively (Figure 5A and Figure S7A). In contrast, African American patients experienced a minor CSS improvement in the period 2009–2013 (HR = 0.92, 95% CI = [0.86, 0.98]) but a dramatic CSS improvement in the most recent period of 2014–2018 (HR = 0.81, 95% CI = [0.75, 0.88]). For Asians and Hispanics, a minor CSS improvement was observed in the two recent 5-year periods compared with 2004–2008. Taken together, we argue that the reduced racial disparities in prostate cancer survival can be explained by the differential survival improvement among each race, especially the rapid CSS improvement in African American patients in 2014–2018.

Compared with patients diagnosed in 2004–2008, all three income groups experienced a significant improvement in CSS survival. However, the high-income group experienced more survival improvement than the other two income groups (Figure 5B and Figure S7B). Specifically, in 2014–2018, the high-income group experienced a substantial CSS improvement, with an adjusted HR = 0.77 (95% CI = [0.72, 0.83]) with respect to the period 2004–2008, compared with an adjusted HR = 0.86 (95% CI = [0.80, 0.91]) for the low-income group and an HR = 0.90 (95% CI = [0.85, 0.96]) for the intermediate-income group. Such a difference might explain the increased socioeconomic disparities observed in recent years.

## 4. Discussion

Using the SEER prostate cancer data for patients diagnosed between 2004 and 2018, we investigated the changes in racial and socioeconomic disparities in survival during the past 15 years. Consistent with previous reports, African Americans and Asians were, respectively, associated with significantly worse and better CSS compared with non-Hispanic whites after adjusting for age, cancer stage, and income. More importantly, we observed an interesting trend: the racial disparities fluctuated in the period of 2004–2011. In the period of 2014–2018, racial disparities in survival were continuously reduced, which was largely driven by the differential survival improvement in prostate cancer patients between different races. A significant survival improvement was observed in non-Hispanic whites, African Americans, and Asians but not in Hispanics during the period of 2014–2018 compared with 2004–2008. Compared with non-Hispanic whites, African Americans achieved a greater survival improvement, while Asians showed less survival improvement. These results can potentially be attributed to a variety of factors, such as improved healthcare access, enhanced early-screening practices, and advancements in treatment approaches. However, this information is limited in the SEER database. Further investigations will be needed to comprehensively analyze the underlying reasons for this trend. Such efforts will also be helpful in reducing the racial disparity in prostate cancer.

The SEER data also confirmed previously reported socioeconomic disparities: prostate cancer patients with high income tend to have better CSS than those with low income. We observed that socioeconomic disparities in survival continuously increased during 2004–2018, indicating the increased benefit for prostate cancer patients with higher income. In fact, the CSS improvement in high-income patients from the period of 2004 to 2008 to the period of 2014 to 2018 was much greater than low-income patients.

Compared with other races, African American patients were more likely to be diagnosed with prostate cancer at a younger age. In addition, African American patients were more likely to be diagnosed with metastatic cancer (stage IV) than non-Hispanic white patients. However, the proportion of patients diagnosed with stage IV cancer was even higher in Asians and Hispanics. Therefore, the poor survival of African American patients and the more favorable survival of Asian patients cannot be explained by their cancer stage at the time of diagnosis, nor can it explain the more substantial survival improvement in African American patients from 2014 to 2018. In all likelihood, the rapid survival improvement in African American in recent years was caused by improved treatment efficacy.

A major limitation of this study is the lack of treatment regimens in the SEER 18 registries’ data. Therefore, our analysis was unable to adjust the influence of treatments on patients’ prognosis. Moreover, some known important prognostic factors are also limited in the SEER data. For example, PSA levels and Gleason scores were known for associations with inferior clinical outcomes and were often considered for treatment decisions. These factors were only available in patients diagnosed after 2009. In addition, SEER data do not include lifestyle variables such as alcohol consumption and smoking, so potential confounding effects of these variables on our results cannot be ruled out.

## 5. Conclusions

In conclusion, SEER analysis revealed significant racial and socioeconomic disparities in prostate cancer diagnosis and survival. While racial disparities dramatically narrowed in the period of 2014–2018, the disparities between high- and low-income patients widened substantially. To further improve prostate cancer survival, more efforts should be implemented to improve the treatment regimens for low-income patients.

## Data Availability

The data analyzed in this study were obtained from the Surveillance, Epidemiology, and End Results (SEER) Program at https://seer.cancer.gov/data/ (accessed on 6 March 2022).

## Supplementary Materials

The following supporting information can be downloaded at: https://www.mdpi.com/article/10.3390/cancers15153977/s1, Table S1: Patient characteristics of the SEER-Prostate cancer cohort; Figure S1: CONSORT flow diagram of this study.; Figure S2: Adjusted hazard ratios of clinical characteristics for CSS in 2009+ patients (A), OS in all patients (B) and OS in 2009+ patients (C); Figure S3: Racial disparities in different patient subgroups diagnosed after 2009; Figure S4: Socioeconomic disparities in different patient subgroups diagnosed after 2009; Figure S5: The change in racial and socioeconomic disparity from 2010 to 2018; Figure S6: The change of age and tumor stage in different race and socioeconomic groups from 2004 to 2018; Figure S7: Survival improvements in different race and socioeconomic groups (after 2009).

## References

[1] Siegel R.L., Miller K.D., Fuchs H.E., Jemal A. (2022). Cancer Statistics, 2022. CA Cancer J. Clin..

[2] Chowdhury-Paulino I.M., Ericsson C., Vince R., Spratt D.E., George D.J., Mucci L.A. (2022). Racial Disparities in Prostate Cancer among Black Men: Epidemiology and Outcomes. Prostate Cancer Prostatic Dis..

[3] Benjamins M.R., Hunt B.R., Raleigh S.M., Hirschtick J.L., Hughes M.M. (2016). Racial Disparities in Prostate Cancer Mortality in the 50 Largest US Cities. Cancer Epidemiol..

[4] Kaur D., Ulloa-Pérez E., Gulati R., Etzioni R. (2018). Racial Disparities in Prostate Cancer Survival in a Screened Population: Reality versus Artifact. Cancer.

[5] Ellis L., Canchola A.J., Spiegel D., Ladabaum U., Haile R., Gomez S.L. (2018). Racial and Ethnic Disparities in Cancer Survival: The Contribution of Tumor, Sociodemographic, Institutional, and Neighborhood Characteristics. J. Clin. Oncol..

[6] Jinna N., Jovanovic-Talisman T., LaBarge M., Natarajan R., Kittles R., Sistrunk C., Rida P., Seewaldt V.L. (2022). Racial Disparity in Quadruple Negative Breast Cancer: Aggressive Biology and Potential Therapeutic Targeting and Prevention. Cancers.

[7] Siddharth S., Sharma D. (2018). Racial Disparity and Triple-Negative Breast Cancer in African-American Women: A Multifaceted Affair between Obesity, Biology, and Socioeconomic Determinants. Cancers.

[8] Cancers|Free Full-Text|Epigenetic Determinants of Racial Disparity in Breast Cancer: Looking beyond Genetic Alterations. https://www.mdpi.com/2072-6694/14/8/1903.

[9] Taparra K., Ing B.I., Ewongwo A., Vo J.B., Shing J.Z., Gimmen M.Y., Keli‘i K.M.K., Uilelea J., Pollom E., Kidd E. (2023). Racial Disparities in Brachytherapy Treatment among Women with Cervical and Endometrial Cancer in the United States. Cancers.

[10] Baliga S., Yildiz V.O., Bazan J., Palmer J.D., Jhawar S.R., Konieczkowski D.J., Grecula J., Blakaj D.M., Mitchell D., Henson C. (2023). Disparities in Survival Outcomes among Racial/Ethnic Minorities with Head and Neck Squamous Cell Cancer in the United States. Cancers.

[11] Wang F., Shu X., Pal T., Berlin J., Nguyen S.M., Zheng W., Bailey C.E., Shu X.-O. (2022). Racial/Ethnic Disparities in Mortality Related to Access to Care for Major Cancers in the United States. Cancers.

[12] DeSantis C.E., Miller K.D., Goding Sauer A., Jemal A., Siegel R.L. (2019). Cancer Statistics for African Americans, 2019. CA Cancer J. Clin..

[13] Dess R.T., Hartman H.E., Mahal B.A., Soni P.D., Jackson W.C., Cooperberg M.R., Amling C.L., Aronson W.J., Kane C.J., Terris M.K. (2019). Association of Black Race with Prostate Cancer-Specific and Other-Cause Mortality. JAMA Oncol..

[14] Iyengar S., Hall I.J., Sabatino S.A. (2020). Racial/Ethnic Disparities in Prostate Cancer Incidence, Distant Stage Diagnosis, and Mortality by U.S. Census Region and Age Group, 2012–2015. Cancer Epidemiol. Biomark. Prev..

[15] Torre L.A., Sauer A.M.G., Chen M.S., Kagawa-Singer M., Jemal A., Siegel R.L. (2016). Cancer Statistics for Asian Americans, Native Hawaiians, and Pacific Islanders, 2016: Converging Incidence in Males and Females. CA Cancer J. Clin..

[16] Zhu Y., Mo M., Wei Y., Wu J., Pan J., Freedland S.J., Zheng Y., Ye D. (2021). Epidemiology and Genomics of Prostate Cancer in Asian Men. Nat. Rev. Urol..

[17] Mahal B.A., Chen Y.-W., Muralidhar V., Mahal A.R., Choueiri T.K., Hoffman K.E., Hu J.C., Sweeney C.J., Yu J.B., Feng F.Y. (2016). National Sociodemographic Disparities in the Treatment of High-Risk Prostate Cancer: Do Academic Cancer Centers Perform Better than Community Cancer Centers?. Cancer.

[18] Helgstrand J.T., Røder M.A., Klemann N., Toft B.G., Brasso K., Vainer B., Iversen P. (2017). Diagnostic Characteristics of Lethal Prostate Cancer. Eur. J. Cancer.

[19] Darcey E., Pereira G., Salter A., Fritschi L., Leavy J., Ambrosini G.L., Boyle T. (2019). The Impact of Lifestyle-Related Factors on Survival After a Prostate Cancer Diagnosis. Eur. Urol..

[20] Hoffmann T.J., Passarelli M.N., Graff R.E., Emami N.C., Sakoda L.C., Jorgenson E., Habel L.A., Shan J., Ranatunga D.K., Quesenberry C.P. (2017). Genome-Wide Association Study of Prostate-Specific Antigen Levels Identifies Novel Loci Independent of Prostate Cancer. Nat. Commun..

[21] Coughlin S.S. (2020). A Review of Social Determinants of Prostate Cancer Risk, Stage, and Survival. Prostate Int..

[22] Rundle A., Neckerman K.M., Sheehan D., Jankowski M., Kryvenko O.N., Tang D., Rybicki B.A. (2013). A Prospective Study of Socioeconomic Status, Prostate Cancer Screening and Incidence among Men at High Risk for Prostate Cancer. Cancer Causes Control.

[23] Major J.M., Norman Oliver M., Doubeni C.A., Hollenbeck A.R., Graubard B.I., Sinha R. (2012). Socioeconomic Status, Healthcare Density, and Risk of Prostate Cancer among African American and Caucasian Men in a Large Prospective Study. Cancer Causes Control.

[24] Clegg L.X., Reichman M.E., Miller B.A., Hankey B.F., Singh G.K., Lin Y.D., Goodman M.T., Lynch C.F., Schwartz S.M., Chen V.W. (2009). Impact of Socioeconomic Status on Cancer Incidence and Stage at Diagnosis: Selected Findings from the Surveillance, Epidemiology, and End Results: National Longitudinal Mortality Study. Cancer Causes Control.

[25] Byers T.E., Wolf H.J., Bauer K.R., Bolick-Aldrich S., Chen V.W., Finch J.L., Fulton J.P., Schymura M.J., Shen T., Van Heest S. (2008). The Impact of Socioeconomic Status on Survival after Cancer in the United States: Findings from the National Program of Cancer Registries Patterns of Care Study. Cancer.

[26] Carpenter W.R., Howard D.L., Taylor Y.J., Ross L.E., Wobker S.E., Godley P.A. (2010). Racial Differences in PSA Screening Interval and Stage at Diagnosis. Cancer Causes Control.

[27] Li X., Sundquist K., Sundquist J. (2012). Neighborhood Deprivation and Prostate Cancer Mortality: A Multilevel Analysis from Sweden. Prostate Cancer Prostatic Dis..

[28] Oake J.D., Harasemiw O., Tangri N., Ferguson T.W., Saranchuk J.W., Bansal R.K., Drachenberg D.E., Nayak J.G. (2021). The Association between Income Status and Treatment Selection for Prostate Cancer in a Universal Health Care System: A Population-Based Analysis. J. Urol..

[29] Surveillance, Epidemiology, and End Results (SEER) Program SEER*Stat Database: Incidence-Based Mortality-SEER Research Plus Data, 18 Registries, November 2020 (2000–2018). http://www.seer.cancer.gov.

[30] Greene F.L., Page D.L., Fleming I.D., Fritz A.G., Balch C.M., Haller D.G., Morrow M. (2002). AJCC Cancer Staging Manual.

[31] Ruhl J., Adamo M., Dickie L. (2016). SEER Program Coding and Staging Manual 2016: Section V.

[32] Ruhl J.L., Callaghan C., Schussler N. (2022). Extent of Disease (EOD) 2018 General Coding Instructions.

