# Supplementary material for: Reduced Racial Disparity as a Result of Survival Improvement in Prostate Cancer
Source: Cancers (Basel). 2023 Aug 4;15(15):3977. doi: 10.3390/cancers15153977 (PMC10417437; doi:10.3390/cancers15153977)
Supplement: Supplementary file 1 [file cancers-15-03977-s001.zip › Supplementary Material.pdf]

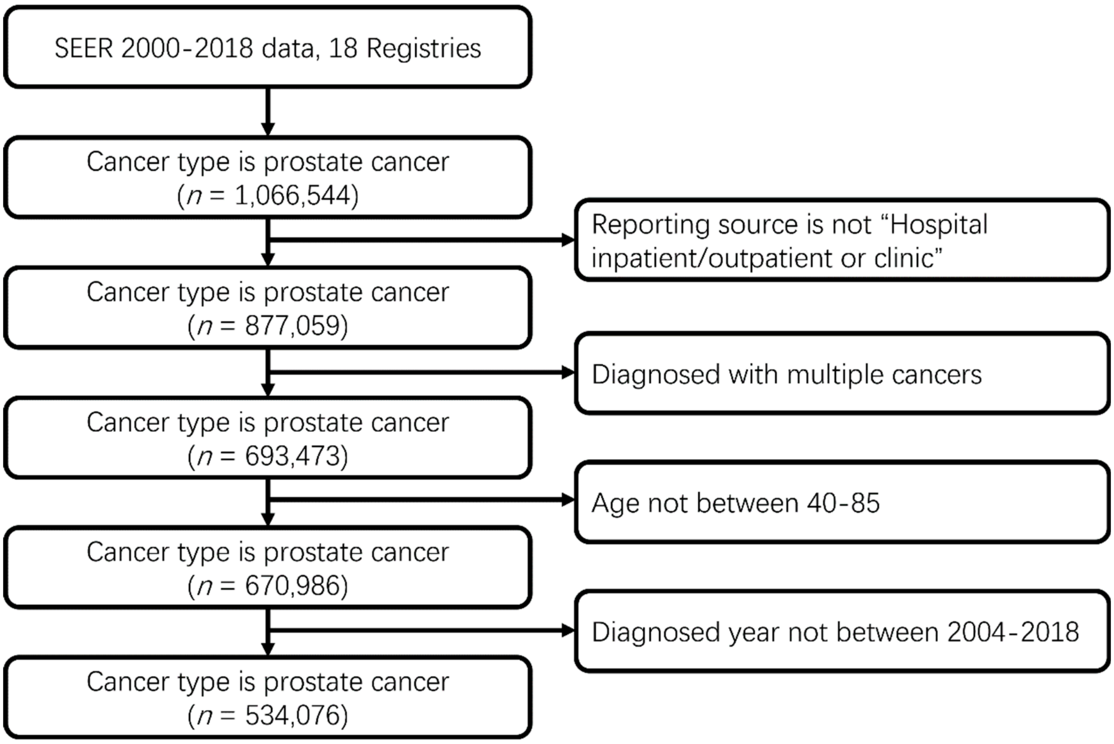

Figure S1. CONSORT flow diagram of this study.

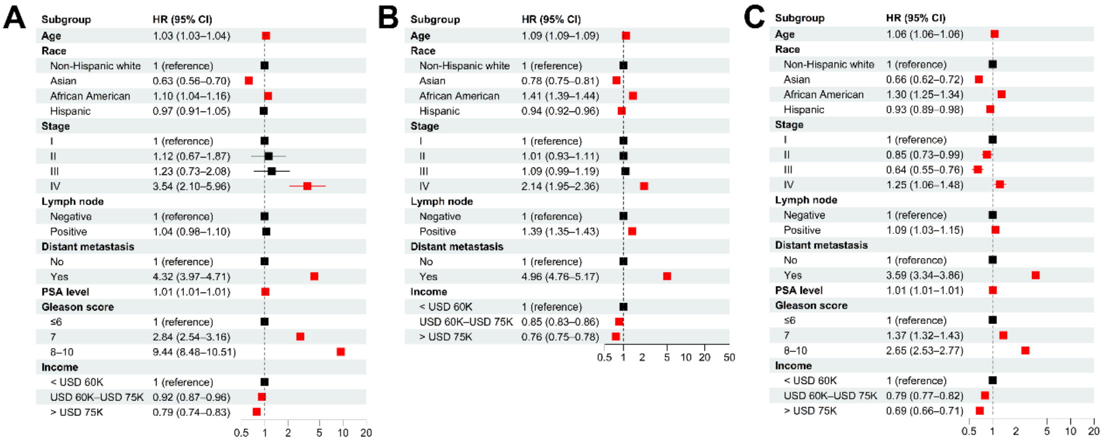

Figure S2. Adjusted hazard ratios of clinical characteristics for CSS in 2009+ patients (**A**), OS in all patients (**B**) and OS in 2009+ patients (**C**). Multivariable Cox models were adjusted for age, race, tumor stage, lymph node involvement, distant metastasis, PSA level, Gleason score and income. 95% CIs were presented in paratheses. Red color indicates a significance level of $p < 0.05$.

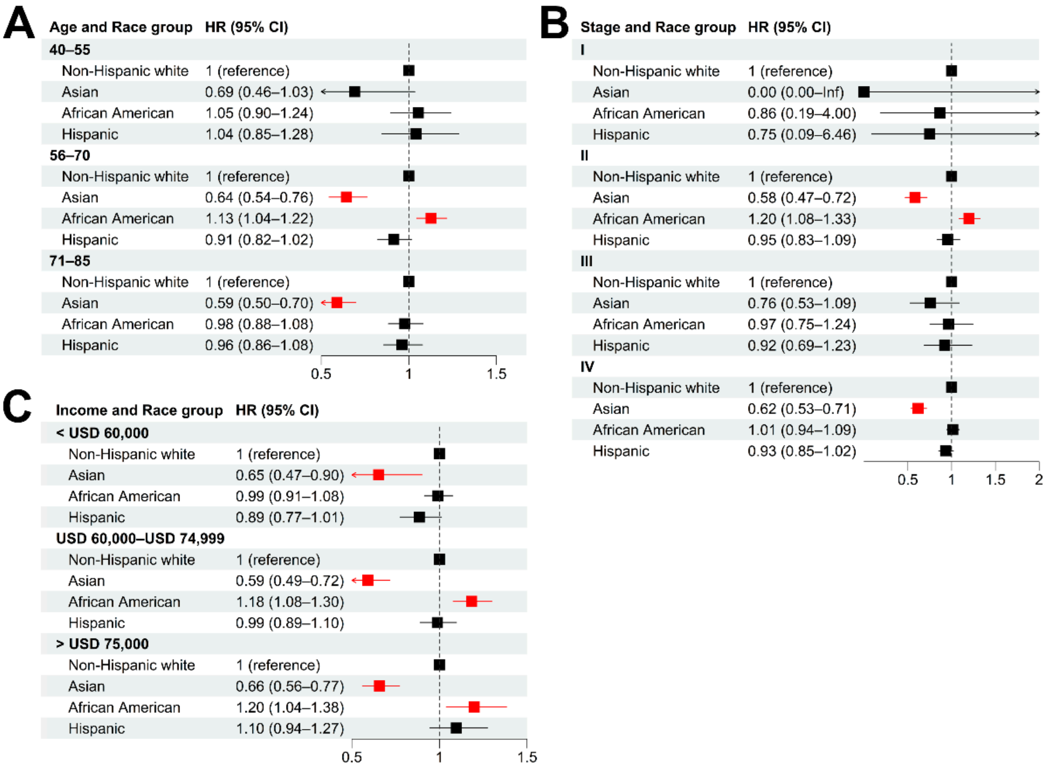

Figure S3. Racial disparities in different patient subgroups diagnosed after 2009. (**A**–**C**): Hazard ratios of race for cancer-specific death in patient subgroups stratified by age (**A**), tumor stage (**B**) and income (**C**) using non-Hispanic white as baseline. Multivariable Cox models were adjusted for age, race, tumor stage, lymph node involvement, distant metastasis, PSA level, Gleason score and income. 95% CIs were presented in paratheses. Red color indicates a significance level of $p < 0.05$.

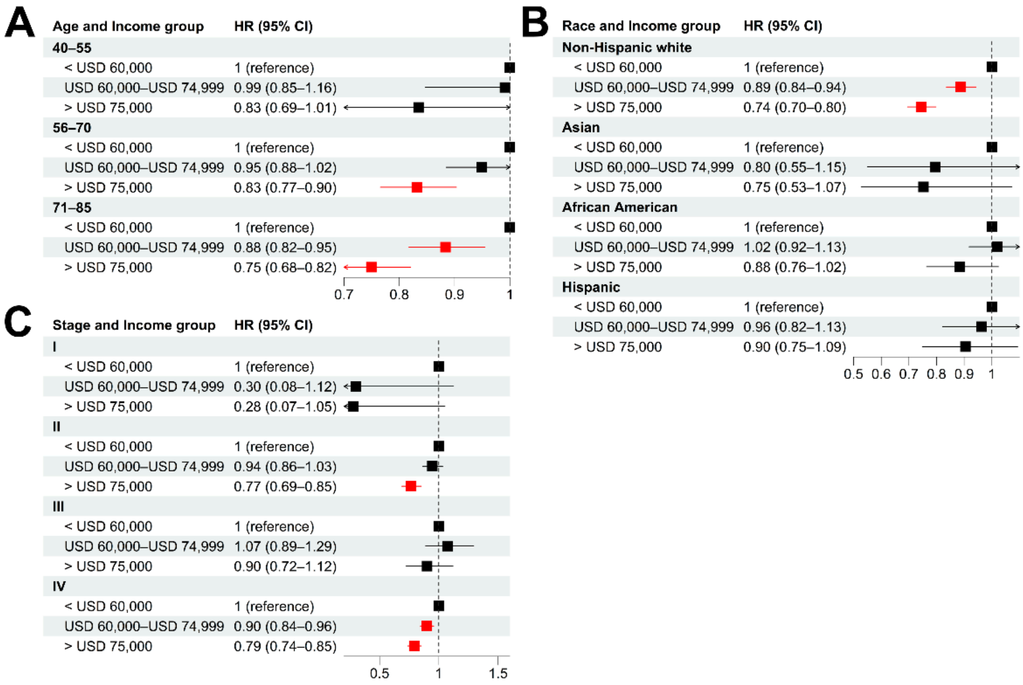

Figure S4. Socioeconomic disparities in different patient subgroups diagnosed after 2009. (**A**–**C**): Hazard ratios of income for cancer-specific death in patient subgroups stratified by age (**A**), race (**B**) and tumor stage (**C**) using low-income (<USD 60,000) group as baseline. Multivariable Cox models were adjusted for age, race, tumor stage, lymph node involvement, distant metastasis, PSA level,

Gleason score and income. 95% CIs were presented in paratheses. Red color indicates a significance level of $p < 0.05$.

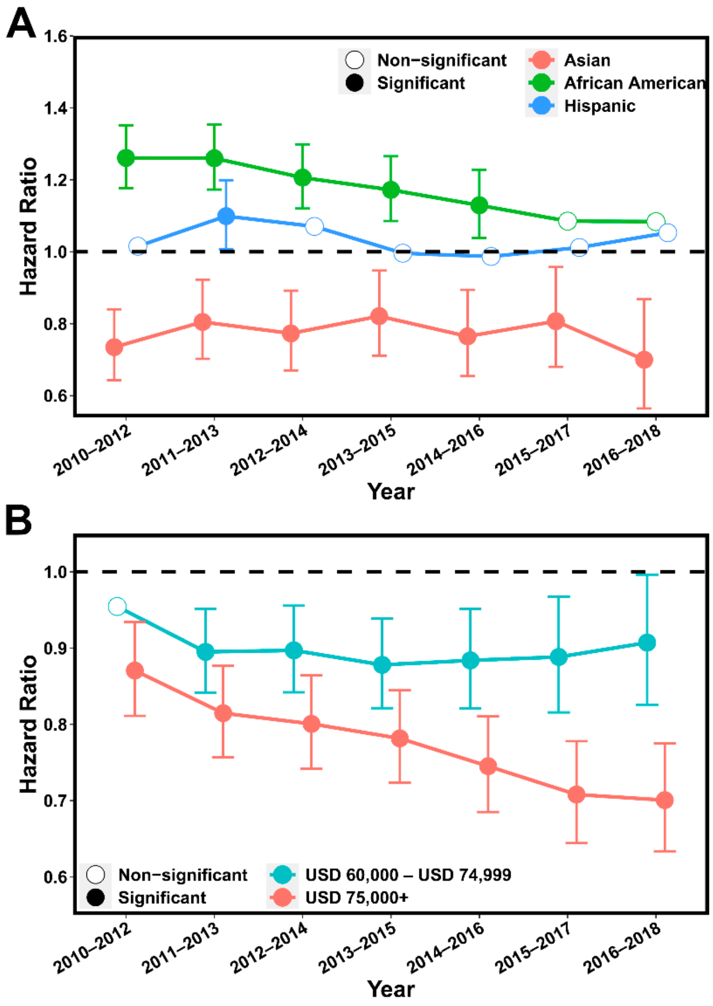

Figure S5. The change in racial and socioeconomic disparity from 2010 to 2018. A: Hazard ratio of Asian, Black and Hispanic for cancer-specific death using non-Hispanic white as baseline. Multivariable Cox models were adjusted for age, tumor stage and income. **B**: Hazard ratio of intermediate-income (USD 60,000–USD 74,999) and high-income (>USD 75,000) groups for cancer-specific death using low-income (<USD 60,000) group as baseline. Multivariable Cox models were adjusted for age, race, tumor stage, lymph node involvement, distant metastasis, PSA level, Gleason score and income. p values below 0.05 were considered significant.

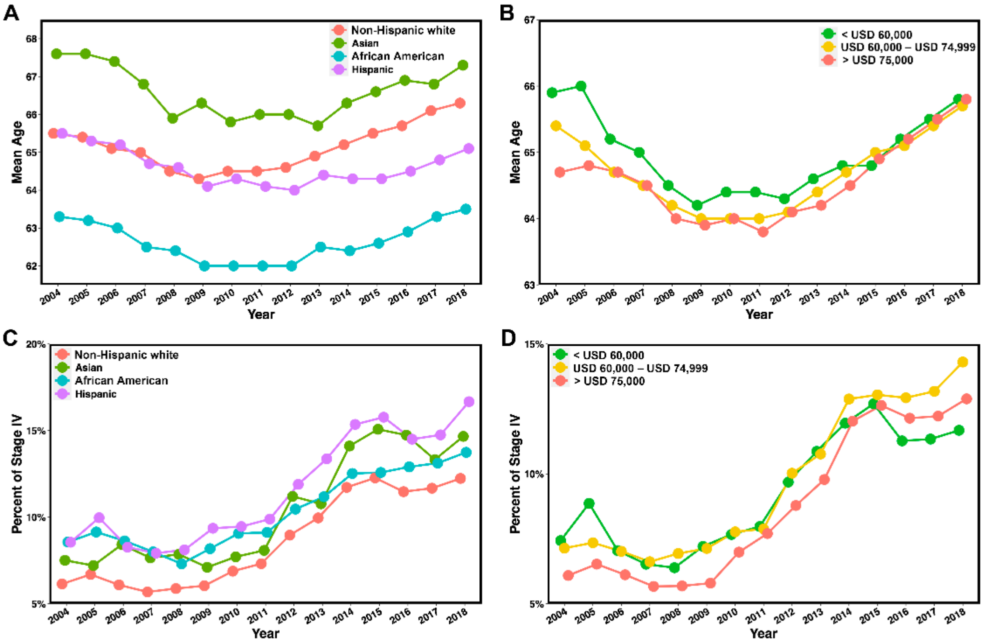

Figure S6. The change of age and tumor stage in different race and socioeconomic groups from 2004 to 2018. A–B: The mean age in the four race groups (**A**) and the three income groups (**B**). (**C–D**) The percentage of stage IV patients in the four race groups (**C**) and the three income groups (**D**). The four race groups contain White, Asian, Black and Hispanic. The three income groups contain low (<USD 60,000), intermediate (USD 60,000–USD 74,999) and high-income (>USD 75,000) groups.

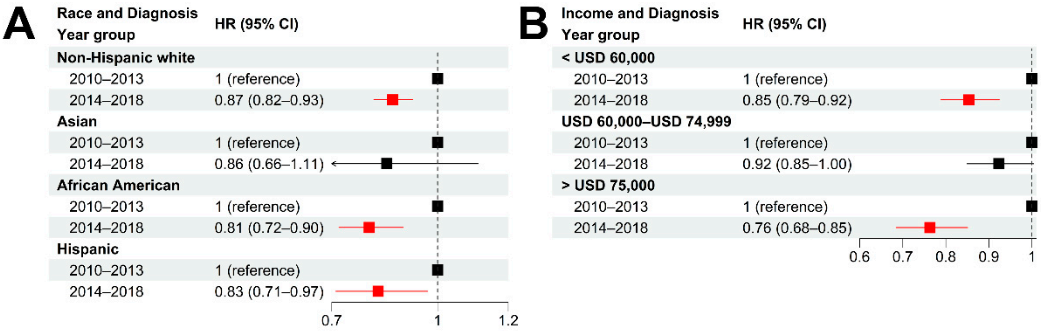

Figure S7. Survival improvements in different race and socioeconomic groups (after 2009). (**A–B**): Hazard ratios of diagnosis year for cancer-specific death in patient subgroups stratified by race (**A**), and income (**B**) using non-Hispanic white and low-income (<USD 60,000) group as baseline, respectively. Multivariable Cox models were adjusted for age, race, tumor stage, lymph node involvement, distant metastasis, PSA level, Gleason score and income. 95% CIs were presented in paratheses. Red color indicates a significance level of $p < 0.05$.
